# mRNA Vaccine: How to Meet the Challenge of SARS-CoV-2

**DOI:** 10.3389/fimmu.2021.821538

**Published:** 2022-01-21

**Authors:** Yingqi Jin, Chen Hou, Yonghao Li, Kang Zheng, Chuan Wang

**Affiliations:** ^1^ Institute of Pathogenic Biology, Hengyang Medical College, University of South China, Hengyang, China; ^2^ Department of Clinical Laboratory, Hengyang Central Hospital, Hengyang, China; ^3^ Hunan Province Cooperative Innovation Center for Molecular Target New Drug Study, University of South China, Hengyang, China

**Keywords:** SARS-CoV-2, COVID-19, mRNA vaccine, challenges, strategies

## Abstract

Coronavirus disease 2019 (COVID-19), caused by severe acute respiratory syndrome coronavirus 2 (SARS-CoV-2) with high infectivity, pathogenicity, and variability, is a global pandemic that severely affected public health and the world economy. The development of safe and effective vaccines is crucial to the prevention and control of an epidemic. As an emerging technology, mRNA vaccine is widely used for infectious disease prevention and control and has significant safety, efficacy, and high production. It has received support and funding from many pharmaceutical enterprises and becomes one of the main technologies for preventing COVID-19. This review introduces the current status of SARS-CoV-2 vaccines, specifically mRNA vaccines, focusing on the challenges of developing mRNA vaccines against SARS-CoV-2, and discusses the relevant strategies.

## 1 Introduction

Severe acute respiratory syndrome coronavirus 2 (SARS-CoV-2) is a novel positive-sense single-stranded RNA coronavirus, which belongs to the *Betacoronavirus* genus (1, 2). Coronavirus disease 2019 (COVID-19) caused by this virus has spread rapidly throughout the world (3, 4), threatening public health and the world economy. Globally, as of December 25 in 2021, more than 278 million confirmed cases of COVID-19, including more than 5 million deaths, have been reported to the World Health Organization (WHO) (5). According to the severity of the disease, COVID-19 is divided into different clinical classifications: asymptomatic, mild, moderate, severe, and critical symptoms (3, 6, 7). Of note, SARS-CoV-2 has high transmission efficiency for asymptomatic or mild cases (8). The clinical symptoms of COVID-19 include fever, sore throat, dry cough, pneumonia symptoms (with or without hypoxemia), and so on. In addition, the complications of patients with COVID-19, such as acute respiratory distress syndrome, sepsis, acute liver and kidney injury, multisystem inflammatory syndrome, autoimmune hemolytic anemia, and neurological complications, are also worthy of attention (9).

The epidemiological studies of COVID-19 have demonstrated that airborne transmission is the major mode in the spread of SARS-CoV-2. Significantly, fecal–oral transmission and transplacental transmission are possible (10–12). On the other hand, SARS-CoV-2 can spread between humans and animals (13). Recent studies have suggested that severe COVID-19 is not limited to the elderly; children and young adults are also at risk (14, 15). Furthermore, the clinical characteristics vary with age. Altogether, SARS-CoV-2 has high infectivity, pathogenicity, and mutability, making the prevention and control of COVID-19 difficult.

Vaccination is one of the most effective and economical ways to prevent and control infectious diseases. It is essential to develop safe and effective vaccines for implementing mass vaccination to prevent and control the COVID-19 pandemic. In recent years, mRNA vaccines have been studied extensively for the prophylaxis and control of infectious diseases (16–18). Compared with other vaccines like whole bacteria, subunit, and DNA vaccine, the mRNA vaccine can induce T-cell and B-cell immune response and is non-integrating and naturally degradable (19–22). In addition, the fast and simple production procedures, free of eggs and cells, make the mRNA vaccine a promising and attractive vaccine candidate that potentially fills the gap between the emerging epidemic and the urgent need for effective vaccines (23). So far, it has been demonstrated that BNT162b2 (BioNTech/Pfizer) and mRNA-1273 (Moderna), granted authorization for emergency use, are generally safe and efficacious to prevent COVID-19 (24–26).

Worldwide, despite the measures taken to control COVID-19 disease spread, infections still occur, suggesting that more effective vaccines will be an immediate need to end the pandemic. Here, we focus on the current challenges of the mRNA vaccine against SARS-CoV-2 and discuss possible countermeasures to contain the continuing spread of the disease.

## 2 Challenges

With the continued global epidemic, the COVID-19 mRNA vaccines still face several important challenges: SARS-CoV-2 variants, protective immunity, immune evasion, vaccinated population, and adverse reactions after vaccination. Undoubtedly, more in-depth knowledge on these challenges will contribute to the design of safer and more effective mRNA vaccines in the future.

### 2.1 Variants

Generally, the RNA virus has a high mutation rate. In addition, all viruses, including SARS-CoV-2, may change over time (27, 28). Most changes have little impact on the properties of the virus. However, some changes may affect the properties of the virus reported to the WHO, such as transmission capacity, related disease severity, or the performance of vaccines, therapeutic medicines, diagnostic tools, and so on (29). For SARS-CoV-2, multiple selective pressure may develop novel variants. It is considered that the most likely selective pressure is to increase the inherent adaptability of the virus by directly replicating within the host or spreading between the hosts (30, 31). In addition, variants may include mutations that change interactions with key host components (32). Of note, selective pressure can induce mutations that permit variants to escape from adaptive immune responses (30).

#### 2.1.1 Antibody Neutralization and Immune Evasion

In the changing global SARS-CoV-2 pandemic context, several variants including B.1.1.7 (Alpha), B.1.351 (Beta), B.1.617.2 (Delta), etc. showed different sequence variations and amino acid sequence changes of the spike protein, which has been reported and has aroused wide concern due to their widespread transmission and possible immune evasion (33).

For the Lambda variant, the receptor-binding domain (RBD) mutation can weaken the ability to recognize broadly neutralizing antibodies. Mutations of T76I and L452Q induce higher infectiousness (34). Besides, the 7-amino-acid deletion within the N-termini domain outside the RBD domain leads to immune evasion. Notably, antibodies from Pfizer-BioNTech vaccinated individuals drop about 3.5-fold for the Delta variant after 6 months (35). The Delta variants have the characteristics of several spike protein mutations, which may affect immune responses and vaccine potency, including T19R, Δ157-158, L452R, D950N, T478K, P681R, and D614G (36, 37). Significantly, SARS-CoV-2 strains with mutations at P681R may enhance viral replication, resulting in higher viral loads and increased spread (38). Also, laboratory data suggested that D614G substitution, which is prevalent among SARS-CoV-2 strains, improves the competitive fitness, infectivity, and transmission of the strains in primary human cells and animal models (31, 39). Besides, the reorganized receptor-binding interface of the Delta variant attenuates interactions with some neutralizing antibodies, resulting in immune evasion (36).

As an important strategy to control the COVID-19 pandemic, mRNA vaccination remains effective in preventing symptomatic and severe COVID-19 associated with infection from several variants. However, whether the effectiveness of mRNA vaccines will be reduced due to variants is still a concern. Using a TNCC (a test negative case control) analysis, the estimated vaccine effectiveness against symptomatic disease with Delta infection for two doses of BNT162b2 is approximately 88% (95% CI, 85.3 to 90.1) (40). Due to concerns about thrombotic events after vaccination with ChAdOx1 nCov-19, heterologous mRNA boost strategies have been recommended and implemented (41, 42). Laboratory data have indicated that heterologous BNT162b2 boost after ChAdOx1 nCov-19 consistently leads to higher neutralizing titers against the Alpha, Gamma, and Beta variants compared with homologous BNT162b2 vaccination. Interestingly, homologous BNT162b2 prime boost seems to be more efficient in generating neutralizing antibodies against the Delta variant (43, 44). Similarly, it has been reported that an mRNA-1273 boost can induce the production of antibodies that neutralize the B.1.351 variant. In addition, mRNA-1273 vaccine can stimulate the SARS-CoV-2-specific memory B cell produced by the first dose of ChAdOx1 nCov-19 vaccine. Compared with a ChAdOx1 nCov-19 boost, an mRNA-1273 boost may provide better immune protection against the B.1.351 variant (45).

#### 2.1.2 Vaccine Breakthrough Infections

The major factor of the vaccine breakthrough infections of variants may be obtaining new antigenic properties to circumvent the recognition of broadly neutralizing antibodies generated from vaccination, which may be enhanced by the weakening of immune protection of the vaccination over time (35). With the emergency and wide spread of variants and the occurrence of vaccine breakthrough infections, some studies have compared the protective efficiency of mRNA vaccines against SARS-CoV-2 variants of concern, assessing variant-specific viral loads or neutralizing antibodies in cases of vaccine breakthrough infections, to prevent and control the global SARS-CoV-2 pandemic better (46, 47).

A case-to-case study, comparing the effectiveness of mRNA vaccines against Delta versus Alpha variants, has found higher infectivity with the Delta variant infections and significantly higher odds of vaccine breakthrough infections in Delta cases when compared with Alpha cases (47). Additionally, a multicenter retrospective cohort study of patients with B.1.617.2 infection showed that the odds of severe COVID-19 needing supplemental oxygen were significantly lower following vaccination compared with unvaccinated individuals in the vaccine breakthrough group (33). Interestingly, vaccinated and unvaccinated groups at diagnosis have similar PCR cycle threshold values, but viral loads declined faster in vaccinated patients.

Variants may cause adverse effects on protective efficacy of currently available mRNA vaccines. The emergence and transmission of variants present a grand challenge for the prevention and control of the SARS-CoV-2 pandemic *via* mRNA vaccination (48–50). Differences in the number of patients with variant infections, age, individuals receiving mRNA vaccines, follow-up of vaccinated individuals, sensitivity or specificity of PCR testing, and other aspects may have an impact on evaluating the effectiveness of mRNA vaccines (40). Therefore, study protocols and data collection assessing the effectiveness of mRNA vaccines should be carefully designed and implemented. Detecting the SARS-CoV-2 nucleic acid of patients and reviewing the existing variants on neutralizing antibodies are required to understand the transmissibility, infectivity, virulence, and immune escape of variants. Evaluating the effectiveness of current mRNA vaccines against SARS-CoV-2 will contribute to develop elaborate strategies for more effective mRNA vaccines against the COVID-19 pandemic.

### 2.2 Protective Immunity

Although much remains to be determined about immune-related factors that protect against SARS-CoV-2 infection, emerging data have demonstrated the importance of humoral and cellular immunity in terms of protection (51, 52). Therefore, it is important to ensure the development of effective mRNA COVID-19 vaccines.

Generally speaking, mRNA vaccines have the unique ability to induce the innate immune system to regulate antigen-specific immune responses. mRNA vaccines can be identified by MHC I and MHC II, which led to antigen-specific humoral and cellular immune responses (53). Meanwhile, it has adjuvant features which can timulate immune cells to secrete tumor necrosis factor-α (TNF-α), interferon-α (IFN-α), and other cytokines (CK) that activate the consuming adaptive immune responses (54). Besides, the mRNA vaccine produces high levels of virus-blocking antibodies, known as neutralizing antibodies (nAb), so many scientists believe that it is superior to other vaccines in preventing infection (55). However, the results of several mRNA candidate vaccine studies show that mRNA may stimulate excessive immune response, which will stimulate cells to secrete large amounts of interferon, thereby inhibiting the effect of mRNA translation and ultimately leading to immune response termination (56, 57). In addition, there is currently a lack of information on the protective life span caused by mRNA vaccines. Effective measurements of vaccines and definitive experimental descriptions of duration do not yet exist. Experience with other human coronaviruses has shown that reinfection is possible due to reduced antibody response. Special attention must be paid to mRNA potential problems, which will help us use mRNA more effectively (58, 59).

In the absence of data for humans, animal studies can help to identify potential correlates of protection. However, there is still a lack of animal models that can fully simulate human immune responses, and animal studies cannot fully predict efficacy in humans (60). The results of H10N8 and H7N9 mRNA vaccines showed that mRNA vaccines could stimulate the human immune response to produce higher neutralizing antibodies, but the animal immune response could only produce lower neutralizing antibodies (21), which proved this point of view. Moreover, since SARS-CoV-2 is a novel pathogen, any surrogate endpoints identified in animal studies would ideally need validation in clinical trials. Researchers need to continue to seek clinical evidence of the effectiveness of mRNA vaccines in human studies (24).

### 2.3 Vaccinated Population

As we all know, the immune system is related to multiple factors which obviously have roles both in innate and adaptive immunity. The monitoring of protective immunity in different immunized populations with vaccination is one of the most important factors in the effectiveness of vaccine. The physical conditions of the recipient as well as sex and age also affect the effectiveness of vaccine.

SARS-CoV-2 is extremely infectious. Everyone can be considered a susceptible group, and the infected individuals present a variety of symptoms (61). Some initial observations have indicated that adults are more likely to contact SARS-CoV-2 than children (62); however, limitation in testing availability in many countries during the pandemic has made it difficult to accurate quantify the true risk of infection in individuals. It is certain that patients with other clinical diseases may be more susceptible to infection (63). Recently, the WHO has shown that young people are becoming the main spreader of SARS-CoV-2, and people under 40 are more susceptible to infection, which will increase the risk of infection for the most vulnerable groups, especially the elderly and patients (64). Additional clinical trials are needed to better establish the infection rate of SARS-CoV-2.

Vaccines have made a huge contribution in preventing infection. It is crucial to understand the difference between the antibody response of different immune populations to vaccination and infection as soon as possible. Compared with traditional vaccines, there are unique advantages of the use of mRNA-based antiviral vaccines (65). The mRNA-based vaccines mRNA-1273 and BNT162b1 are two mRNA vaccines that are currently progressing rapidly. The phase 3 trial of mRNA-1273 (the fastest-growing mRNA vaccine currently in development, which stimulates the expression of a target antigen after vaccination) was launched in late July 2020. The trial was mainly for people who have no infection history at the age of 18 or older and included some persons with different racial and ethnic backgrounds. The results have shown that the safety of the mRNA-1273 vaccine is 94.1% effective in preventing SARS-CoV-2 when an individual is completely vaccinated (25). Infection and the safety of mRNA-1273 are not affected by age (66). However, the trial did not precisely assess the immunogenicity of different populations, and pregnant women and children were not included in the trial. Additional evaluation of vaccines needs to be planned (25, 67). Consistent with mRNA-1273, the BNT162b2 vaccine showed 94% efficacy in preventing SARS-CoV-2 infection, while there is still controversy about the immunization effect in different populations (68, 69). Taken together, although the mRNA vaccine under development provides great hope for the prevention of COVID-19, there is still a lack of strong proof of the immune effect in different populations. More in-depth research should be conducted to understand the vaccination status in different people. Understanding the mechanisms involved in individual disparity in the effectiveness of mRNA vaccines will contribute to improve the development of mRNA vaccines.

### 2.4 Adverse Reactions

As of December 21, 2021, there are over 300 COVID-19 vaccine candidates being developed. Of these, at least 137 candidates derived from different platforms, including the whole-microbe approach (inactivated vaccine, live-attenuated vaccine, and viral vector vaccine), the subunit approach, and the genetic approach (nucleic acid vaccine), are currently in clinical development (70, 71). Like with any vaccine, mild to moderate side effects may emerge after being vaccinated against COVID-19 (72). It is a normal sign of the immune response of the body to the vaccine (73). However, severe side effects or adverse reactions could be experienced, causing fear and anxiety about vaccination in the population (72, 74). Therefore, evaluating safety is more conducive to the development and emergency use of COVID-19 vaccines.

Theoretically, mRNA vaccine has greater security compared with other types of vaccines, for instance, without infectious virus in the production process, a lower risk of virulence reversion, and insertional mutagenesis (75). Nevertheless, high-quality real-world safety data on mRNA-based COVID-19 vaccines are still relatively scarce in the literature. Common adverse reactions to mRNA-based COVID-19 vaccines include allergic reaction and some other side effects such as arm pain, fatigue, and a mild fever (**Table 1**). Also, serious adverse reactions following BNT162b2 mRNA COVID-19 vaccination including pericarditis, arrhythmias, deep vein thrombosis, pulmonary embolism, myocardial infarction, intracranial hemorrhage, and thrombocytopenia need more emphasis (82) (**Table 1**). A study demonstrated a possible association between Bell’s palsy and vaccination with BNT162b2 and mRNA-1273 COVID-19 mRNA vaccine (82). Regarding other mRNA vaccines like CVnCov, Arct-021, and LNP-nCovsaRNA, the main adverse reactions are mild (**Table 1**). Clinical trials may be inherently limited in assessing vaccine safety because of the small number of participants and the inadequate representation of the sample. Hence, long-term and comprehensive monitoring of vaccine safety is required.

**Table 1:** The common adverse reactions of mRNA vaccine candidates in clinical trials.

Vaccine name	BNT162b2 (26, 69)	mRNA-1273 (25, 76)	CVnCoV (77, 78)	ARCT-021 (79)	LNP-nCoVsaRNA (80)	ChulaCov19 (81)
Phase	III/IV	IV	III	II	I	I
Developers	BioNTech/Fosun Pharma/Pfizer	Moderna/NIAID	CureVac AG	Arcturus/Duke-NUS	Imperial College London	Chulalongkorn University
Age at vaccination	≥16 years	≥18 years	18–60 years	20–80 years	18–75 years	18–75 years
Route of administration	IM	IM	IM	IM	IM	IM
Number of doses	2	2	2	2	2	2
Duration	The second injection was given after 28 days	The second injection was given after 28 days	The second injection was given after 28 days	–	The second injection was given after 28 days	The second injection was given after 21 days
Time of adverse reaction	Mostly start about 15 min	Mostly start about 15 h after vaccination	Mostly start about 24 h after immunization	–	–	–
Adverse reaction	Right axillary lymphadenopathy; paroxysmal ventricular arrhythmia; right leg paresthesia	Pneumonia; immediate systemic allergic reactions	Moderate headache; injection site pain; moderate headache; transient lymphopenia	ARCT-021 was generally well tolerated; most adverse reactions are mild	Purpura on the skin; soreness of the arms; blockage of a vein; small nerve injury	Pain; tenderness; induration/swelling; ulceration; scabs; hypersensitivity
	The injection site: mild-to-moderate injection site pain					
	Respiratory system: cough; shortness of breath					
	Digestive system: decrease appetite; nausea, vomiting; diarrhea					
	Nervous system: fatigue; lethargy; paralysis; headache; dizziness					
	Systemic reaction: asthenia, malaise					
	Skin: night sweats, hyperhidrosis					

Phase: a stage in the development of mRNA vaccine clinical trials.

IM, intramuscular injection.

We found an interesting fact that adverse reactions appear within different time periods after injection of some vaccines by data analysis. What could be the reason? Allergic reactions caused by vaccination are usually lgE-mediated and occurred within the first 30 min after vaccination (83). Not all immediate reactions associated with vaccines are true allergic reactions (84). The allergic reactions are usually related to vaccine components, which include excipients, inactive ingredients, and liposomal delivery vehicles (84, 85). The possibility that differential composition may make adverse events happen at different temporal stages after vaccination still need to be confirmed. Besides, some studies suggest that the incidence of adverse reactions is associated with dosage (82, 86). Whether dosage has an impact on the occurrence of adverse reactions still need to be explored. Meanwhile, interindividual differences in innate immune system should not be disregarded (87).

It is noteworthy that some differences in the safety of vaccines from various platforms have been recognized. An observational study assessed cases of cerebral vein thrombosis and attributed the rates to four COVID‐19 vaccines: tozinameran (Pfizer‐BioNTech, mRNA vaccine), CX‐024414 (Moderna, mRNA vaccine), ChAdOx1 nCov‐19 (AstraZeneca, chimpanzee adenoviral vector vaccine), and AD26.COV2.S (Janssen, adenoviral vector vaccine). It has been found that cases of cerebral vein thrombosis events (with or without thrombocytopenia) were observed for all these four vaccines. Furthermore, compared with adenoviral vaccines, a lower reporting rate of thrombocytopenia and unusual site thrombosis adverse drug reaction was observed for the mRNA vaccine (88). Moreover, a systematic review shows that adverse events associated with serious metabolic, immune system, musculoskeletal, and renal disorders were seen more often with inactivated vaccine recipients; the occurrence of serious gastrointestinal complications and infections was more frequent among viral vector and inactivated vaccine recipients than mRNA vaccines; and serious vessel disorders were observed more in mRNA vaccine recipients (89). Research has found that the common adverse events of ZF2001 (Longcom, a recombinant protein subunit vaccine) were mild or moderate, including injection site pain, swelling, redness, fever, and fatigue. The incidence of fever and fatigue was lower among ZF2001 vaccine recipients than mRNA-based vaccines or adenovirus-vectored vaccines (90). Similarly, the first-in-human study of ZyCoV-D (Cadila Healthcare Limited, DNA vaccine) shows that adverse reactions following vaccination including systemic symptoms (headache, fever, fatigue, nausea, vomiting, diarrhea, arthralgia, and muscle pain) and local reactions (injection site pain and pruritus) were mild to moderate in severity (91).

However, these evaluations of the safety profile of COVID-19 vaccines based on different platforms may have several limitations which are related to sample size, comparator group, follow-up time, age, gender, and ethnic group of vaccinated persons. Further exploring the exact associations between vaccination and associated adverse events will lead to a greater and more comprehensive safety assessment of COVID-19 vaccine candidates. Understanding how serious or life-threatening adverse events could be is helpful to avoid the potential risk of vaccination for patients with related diseases.

## 3 Possible Strategies

Since the outbreak of the COVID-19 pandemic, scientists around the world have been together seeking for a longer-term vaccine solution in order to reduce the risk of SARS-CoV-2 transmission as well as lower COVID-19 morbidity and mortality. Despite significant progress and promising results being gained, the current COVID-19 mRNA vaccine still faces the above intractable challenges. Significantly, the selection of antigen and routes of administration, influencing vaccine efficacy and security, should be taken into account when finding some effective vaccine solution strategies.

### 3.1 Antigen Selection

The core principle of mRNA vaccine is to deliver the mRNA sequence encoding target antigen into the host cell cytoplasm and translate the corresponding antigen by using the host cell machinery, thereby inducing immune responses for the prevention or treatment of disease (85, 92). Some features of antigen, such as immunogenicity and specificity, have an impact on the effectiveness of the mRNA vaccine (18). Therefore, the selection of antigens plays an essential role in designing and developing an mRNA vaccine.

As one of the major structural proteins of SARS-CoV-2, the S (spike) protein plays a crucial role in virus entry and infection and contains several B-cell and T-cell epitopes, which can trigger neutralizing antibodies and immune protection (93, 94). Thus, the S protein is considered a dominant antigen candidate of mRNA vaccine against COVID-19 (95). Both the mRNA-1273 vaccine and BNT162b2 encode the prefusion-stabilized full-length spike protein of SARS-CoV-2 and contribute to prevent and control SARS-CoV-2 and its variant infectiona (25, 96). A study (97) established three mRNA vaccine candidates encoding different antigens for COVID-19, showing that only RQ3013-VLP (encodes the S, M, and E proteins to form SARS-CoV-2 virus-like particles) induced humoral and T-cell immune responses in mice, for RQ3012-Spike (encodes the full-length wild-type S) and RQ3013-VLP contain the same amount of S mRNA. Notably, RQ3011-RBD (2 μg RNA/dose, RNA encoding the RBD of the S glycoprotein (residues 331–524) of SARS-CoV-2 with both an N-terminal signal peptide and a C-terminal membrane-anchoring helix) failed to elicit sufficient immunity in mice. However, some improvements may strengthen the immunogenicity of RBD-based mRNA vaccine. Sun et al. (98) found that TF-RBD, an mRNA vaccine based on the trimeric RBD fused to ferritin-formed nanoparticles, induced a stronger humoral immunity response and produced RBD-specific antibodies and neutralizing antibodies as well as Th1-biased cellular response in mice, when compared with T-RBD (an mRNA vaccine based on the trimeric RBD). Furthermore, the mRNA vaccine has a high flexibility, because mRNA(s) encoding single or multiple antigens can be co-delivered to enhance and broaden immune responses (99, 100). The TF-RBD multivalent vaccine targeting SARS-CoV-2 variants (B.1.1.7 and B.1.351) can elicit a broad spectrum of neutralizing antibodies (98). The TF-RBD mRNA vaccine strategy contributes to establish multivalent vaccines against SARS-CoV-2 mutations. Besides the S protein and RBD, other antigens, containing the S1 subunit and N-terminal domain of the S protein, are used in the studies of immunogenicity as antigen candidates for vaccine against COVID-19 (93, 101, 102). However, whether these antigen candidates are suitable for use in mRNA vaccine for preventing and controlling COVID-19 still needs to be evaluated.

### 3.2 Mucosal Vaccination

So far, most of the vaccine candidates, including mRNA vaccine in clinical phase against COVID-19, can select intramuscular administration (IM) to induce both humoral and cellular immune responses for preventing and controlling COVID-19 (103). However, IM seems to only prevent lower respiratory tract infections but fails to elicit sterilizing immunity in the upper airway, because it induces a strong serum IgG reflex but does not elicit epithelial cell IgA responses (58, 104). Generally, the ideal vaccine against SARS-CoV-2 infection through mucosal transmission should be able to elicit not only systemic but also mucosal immune responses (105, 106). Recent research suggested that intranasal administration (IN) can elicit high neutralizing antibody generation, mucosal IgA, and T-cell responses to avoid SARS-CoV-2 infection (107). Besides, Du et al. (108) have found that IN shows an excellent profile in inducing mucosal and humoral immune responses in mice, through comparing the immunological potency induced by IN, IM, and ID (intradermal) administration with an RBD-based SARS-CoV-2 vaccine. Furthermore, IN vaccination has some other unique strengths, including non-invasiveness, easy administration, and self-administration, which is a more cost-effective and efficient way of administration during the COVID-19 pandemic. Interestingly, the self-assembling nanocomplex formulated with cationic cyclodextrin-polyethylenimine 2k conjugate (CP 2k) is a safe and effective delivery vector for intranasal mRNA vaccine, which can overcome the nasal epithelial barrier and enhance the intranasal and paracellular delivery of mRNA encoding antigen and induce strong mucosal and systemic immune responses (109). Thus, IN administration may be an advantageous immunization route of COVID-19 mRNA vaccine.

## 4 Conclusion

Over the past several years, it has become clear that mRNA-based vaccines have promising prophylactic applications. The ongoing mRNA vaccines against SARS-CoV-2 play an important role in maintaining public health, being the most recent example of critically important advancements in the field of mRNA vaccines. Some potential risks, such as the emergence of novel SARS-CoV-2 variants, increased immune evasion, and serious adverse effects, may have an impact on the effectiveness or promotion of current available COVID-19 mRNA vaccines. It is necessary to further improve mRNA-based vaccines. In addition to choosing the dominant antigen, modifying nucleosides and optimizing sequences are also possible approaches to strengthen protein translation expression and immunogenicity. Vaccines targeting multivalent antigens and combined vaccines may be used to strengthen protective immunity. The mRNA-based vaccine combined with other vaccines will most likely be able to deal with COVID-19 mutations. Nevertheless, this would increase the complexity of the vaccine and any changes to these parameters may have implications on vaccine production and the interaction of vaccines may maximize adverse events after administration. Additional research will need to define the variation trend of SARS-CoV-2 and prepare for long-term coexistence of SARS-CoV-2. Furthermore, the optimal immunization route should be selected to enhance the protective efficiency of mRNA vaccine. Besides intramuscular, intranasal immunization might also be advantageous for inducing immune response. Taken together, prior work involving mRNA-based vaccines, together with current studies of COVID-19 mRNA vaccines, provides evidence for the viability of this novel vaccine modality. There is still a lack of effective data to show the duration of mRNA vaccines under the changing epidemic situation, and a more complete understanding of COVID-19 mRNA vaccine protection still needs to be pursued. This review is focused on the challenges and possible development strategies of mRNA vaccine against SARS-CoV-2 and its variants, to provide a theoretical basis for preventing and controlling the COVID-19 pandemic. In summary, efficacy, security, production capacity, and costs need to be carefully evaluated so as to determine whether these strategies for improving mRNA vaccine are feasible and effective.

## Author Contributions

All authors listed have made a substantial, direct, and intellectual contribution to the work and approved it for publication.

## Funding

This work was supported by the Natural Science Foundation of Hunan Province under Grant No. 2021JJ40455, the Hengyang Science and Technology Planning Project under Grant No. 202002042415, the Hunan Provincial Key Laboratory for Special Pathogens Prevention and Control Foundation under Grant No. 2014-5, and the Hunan Province Cooperative Innovation Center for Molecular Target New Drug Study under Grant No. 2015-351.

## Conflict of Interest

The authors declare that the research was conducted in the absence of any commercial or financial relationships that could be construed as a potential conflict of interest.

## Publisher’s Note

All claims expressed in this article are solely those of the authors and do not necessarily represent those of their affiliated organizations, or those of the publisher, the editors and the reviewers. Any product that may be evaluated in this article, or claim that may be made by its manufacturer, is not guaranteed or endorsed by the publisher.
